# The first complete chloroplast genome sequence of *Sterculia foetida* Linnaeus (Malvaceae) and a comparative phylogenetic analysis

**DOI:** 10.1080/23802359.2025.2530712

**Published:** 2025-07-14

**Authors:** Peisi Luo, Lijun Huang, Zhiluo Zhou, Jie Chen, Guangxuan Lin, You Wei, Zhenling Yan, Jing Zhou, Fangnan Kong, Juanjuan Jiang, Xuebin Zhang, Jing Zhao, Jingmei Tang, Meiying Lu, Caixia Zhou

**Affiliations:** a Guangxi South Subtropical Agricultural Sciences Research Institute, Guangxi Academy of Agricultural Sciences, Chongzuo, China; b College of Coastal Agricultural Sciences, Guangdong Ocean University, Zhanjiang, China; c Administration Bureau of Zhanjiang Mangrove National Nature Reserve, Zhanjiang, China

**Keywords:** High-throughput sequencing, plant systematics, gene annotation

## Abstract

*Sterculia foetida* L. (1753), a tree species in the family Malvaceae, is widely cultivated in tropical regions  for its applications in food, medicine, and biodiesel production. This study reports the first complete chloroplast genome sequence of *S. foetida*, which is 162,637 bp long with a GC content of 36.68%, containing a large single-copy region (91,324 bp), a small single-copy region (20,347 bp), and two inverted repeat regions (25,483 bp each). The genome encodes 130 genes: 85 protein-coding genes, eight rRNA genes, and 37 tRNA genes. Phylogenetic analysis shows *S. foetida* is closely related to *S. pexa*.

## Introduction

*Sterculia foetida* L., commonly known by various names such as ‘Java olives’ in English, ‘Sam-rong’ in Thai, ‘Janglibadam’ in Hindi and Bengali, and ‘Gorapubadam’ in Tamil, is widely cultivated in tropical regions of Africa, South America, northern Australia, and southern China (Vipunngeun and Palanuvej 2009; Rahman et al. 2012; Alam et al. 2021). The leaves of this species are clustered at the tips of the branches, palmate in shape, and typically consist of 7–9 leaflets. The inflorescence is apical on the branchlets and paniculate in form. The follicles are ellipsoid, woody, and measure approximately 5–8 cm in length, each containing 10–15 seeds. The seeds are black, ellipsoid, and about 1.5 cm long (Editorial Committee of Chinese Flora 2007).

*S. foetida* is utilized in both food and biodiesel production and shows promising medicinal value in drug delivery systems, as well as in antibacterial and anticancer therapies (Sambasivam and Murugavelh 2019; Peláez et al. 2020; Hadke and Khan 2021; Peláez et al. 2021; da Silva et al. 2021). Similar to *S. foetida*, other species within the genus *Sterculia* also possess significant economic and medicinal potential. For example, the seeds of *S. nobilis* are processed into various food products; *S. villosa* is used to produce a refreshing beverage; *S. lychnophora* is employed in the prevention and treatment of pharyngitis; and the gums of *S. urens* and *S. striata* have applications in drug delivery systems (da Silva et al. 2021; Wang et al. 2024).

Wilkie et al. (2006), Wang et al. (2021), and Liang et al. (2024) conducted phylogenetic analyses of various species within the genus *Sterculia* at different taxonomic levels; however, none of these studies included *S. foetida*. Hamada et al. (2025) analyzed phylogenetic relationships among four *Sterculia* species, including *S. foetida*, by integrating *MatK* gene sequences with morphological characteristics. However, constructing phylogenetic trees based on a single gene may not adequately resolve evolutionary relationships among species. In this study, we present the first complete chloroplast genome sequence of *S. foetida*, providing a valuable genomic resource to clarify its phylogenetic relationship with other species in the family Malvaceae.

## Materials and methods

**Figure 1.**
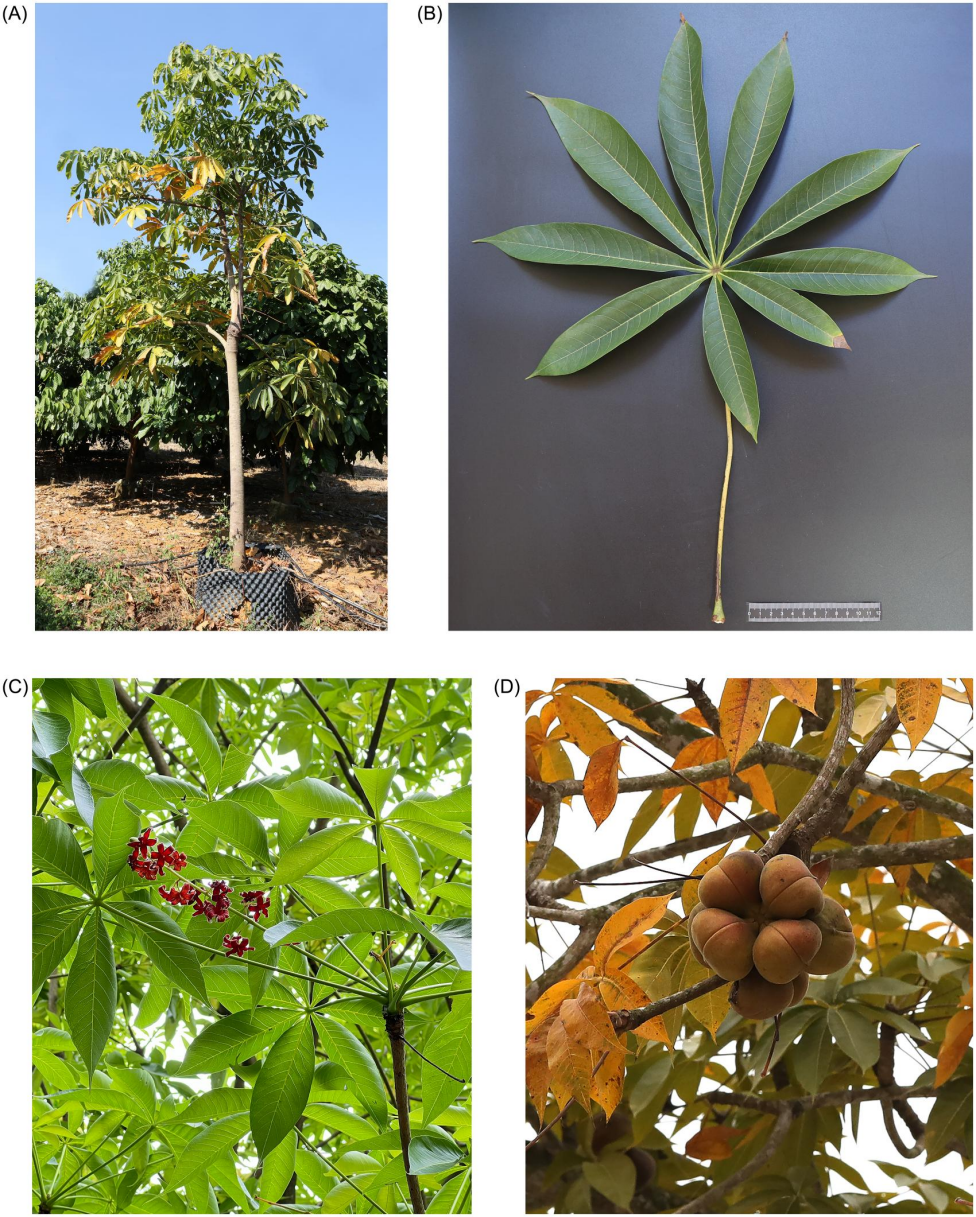
Morphological characteristics of *S. foetida.* (A) Whole plant. (B) Leaf. (C) Inflorescence. (D) Follicle. Photographs were taken by Peisi Luo and Guangxuan Lin, with no copyright restrictions.

Fresh *S. foetida* leaf samples were collected from Chongzuo City, Guangxi Province, China (106.79505° E, 22.34618° N) (Figure 1). Each sample weighed approximately 100 mg, and three biological replicates were obtained. A voucher specimen was deposited in the herbarium of the Guangxi South Subtropical Agricultural Sciences Research Institute under voucher number 2024Sf001 (contact: Peisi Luo, luo049249@gmail.com). DNA was extracted using the improved CTAB method (Doyle 1987). Complete chloroplast genome sequencing was performed using the Illumina NovaSeq 6000 platform (Illumina, San Diego, CA) by Shenzhen Huitong Biotechnology Co. (Shenzhen, China). Raw reads were filtered using the NGS QC Toolkit v2.3.3 (Patel and Jain 2012), and the resulting high-quality paired-end reads were merged. Genome assembly and gene annotation were carried out using SPAdes v3.11.0 and PLANN software, respectively (Zheng et al. 2020). The number and types of annotated genes were recorded, and the chloroplast genome map was generated using OGDRAW software.

To investigate the phylogenetic position of *S. foetida*, the complete chloroplast genomes of seven *Sterculia* species were downloaded from NCBI. According to the findings of Wang et al. (2021), 10 additional species from the closely related genera *Firmiana* and *Heritiera* were also included in the phylogenetic analysis. Two species from the families Fabaceae and Dipterocarpaceae were selected as outgroups. All species in the analysis – including the 18 Malvaceae species and the two outgroup taxa – belong to the order Malvales (Stevens 2024). Sequence alignment was performed using MAFFT v7.490 (Katoh and Standley 2013). A maximum-likelihood (ML) phylogeny was constructed using the IQ-TREE web server (http://iqtree.cibiv.univie.ac.at/) with 1000 ultrafast bootstrap replicates. The best-fit substitution model, GTR + F + G4, was selected based on the Bayesian information criterion (Nguyen et al. 2015; Hoang et al. 2018).

## Results

**Figure 2.**
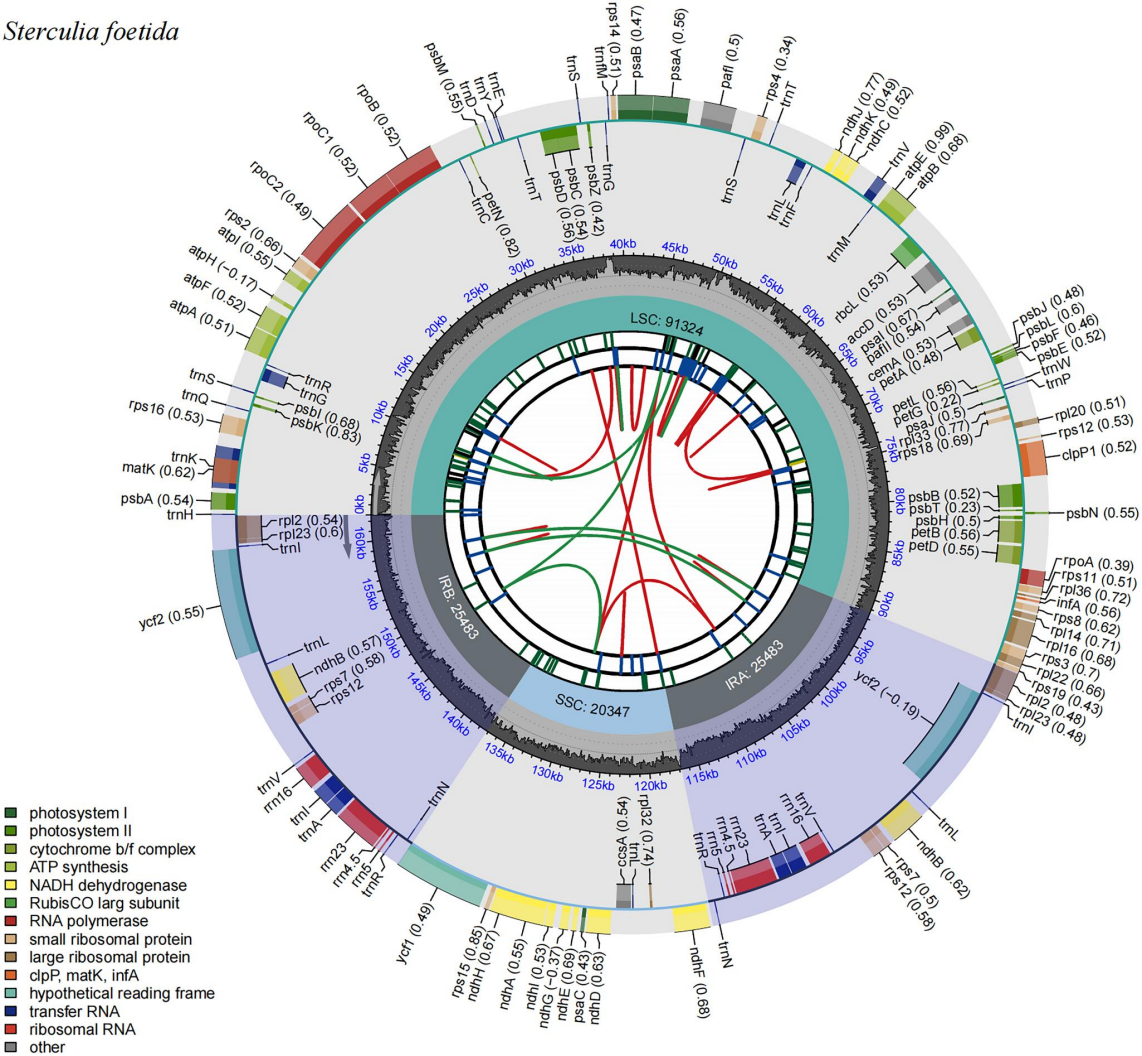
Genomic map of the chloroplast genome of *S. foetida*. The map consists of six tracks arranged from the center outward. The first track represents dispersed repeats, with red arcs indicating direct repeats and green arcs indicating palindromic repeats. The second track displays long tandem repeats marked by short blue bars. The third track shows short tandem repeats (microsatellites) as colored bars. The fourth track indicates the chloroplast genome regions: small single-copy, inverted repeats (IRs), and large single-copy. The fifth track represents the GC content. The outermost track displays coding genes categorized by function. Transcription directions for genes on the inner and outer circles are clockwise and counterclockwise, respectively.

The clean reads were mapped to the chloroplast genome sequence with an average coverage depth of 1404× (Figure S1), indicating that the chloroplast genome of *S. foetida* was accurately assembled (Ni et al. 2023). The complete chloroplast genome of *S. foetida* exhibited the typical quadripartite structure, with a total length of 162,637 bp, comprising a large single-copy region of 91,324 bp, a small single-copy region of 20,347 bp, and two inverted repeats (IRs) of 25,483 bp each. The genome had an overall GC content of 36.68% and encoded 130 genes, including 85 protein-coding genes (PCGs), eight ribosomal RNA genes (rRNAs), and 37 transfer RNA genes (tRNAs) (Figure 2). Introns were identified in 17 genes. Among them, 15 genes (*atpF*, *ndhA*, *ndhB*, *petB*, *petD*, *rpl16*, *rpl2*, *rpoC1*, *rps16*, *trnA-UGC*, *trnG-UCC*, *trnI-GAU*, *trnK-UUU*, *trnL-UAA*, and *trnV-UAC*) contained a single intron, while two genes (*clpP1* and *pafI*) contained two introns. Additionally, 13 genes – *rps16*, *atpF*, *rpoC1*, *pafI*, *clpP1*, *petB*, *petD*, *rpl16*, *rpl2*, *ndhB*, and *ndhA* – were identified as cis-splicing genes (Figure S2), while *rps12* was identified as a trans-splicing gene (Figure S3).

**Figure 3.**
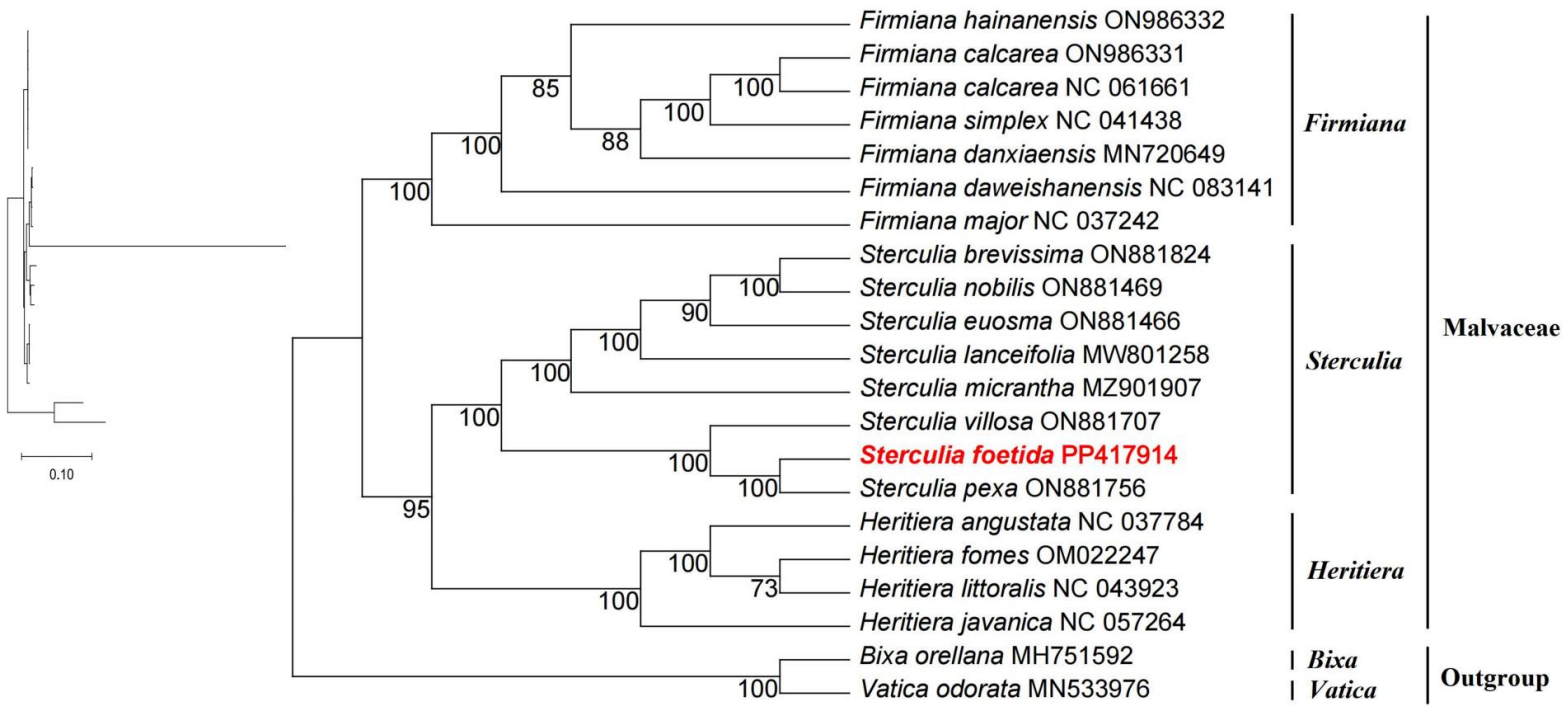
Phylogenetic position of *S. foetida* was inferred using the maximum-likelihood (ML) method based on 20 complete chloroplast genomes, with *Bixa orellana* and *Vatica odorata* used as outgroups. Statistical support values are shown at the nodes. The chloroplast genomes used to construct the phylogenetic tree include: *F. hainanensis* ON986332 (Tan et al. 2023), *F. calcarea* ON986331 (Tan et al. 2023), *F. calcarea* NC061661 (unpublished), *F. simplex* NC041438 (unpublished), *F. danxiaensis* MN720649 (Lin et al. 2020), *F. daweishanensis* NC083141 (Tan et al. 2023), *F. major* NC037242 (Ya et al. 2018), *S. brevissima* ON881824 (Jin et al. 2024), *S. nobilis* ON881469 (Jin et al. 2024), *S. euosma* ON881466 (Jin et al. 2024), *S. lanceifolia* MW801258 (Jin et al. 2024), *S. micrantha* MZ901907 (unpublished), *S. villosa* ON881707 (Jin et al. 2024), *S. pexa* ON881756 (Jin et al. 2024), *H. angustata* NC037784 (Zhao et al. 2018), *H. fomes* OM022247 (Yoocha et al. 2023), *H. littoralis* NC043923 (unpublished), *H. javanica* NC057264 (Zhang et al. 2020), *B. orellana* MH751592 (Dai et al. 2019), and *V. odorata* MN533976 (Wang et al. 2021).

A total of 20 species were included in the phylogenetic analysis, comprising 18 species from the family Malvaceae, with *Bixa orellana* and *Vatica odorata* serving as outgroups. The results revealed that the 20 species clustered into four major groups, with members of the same genus grouping together – particularly those from the genera *Firmiana*, *Sterculia*, and *Heritiera*. The genus *Heritiera* was found to be more closely related to *Sterculia* than to *Firmiana*. Moreover, among the eight *Sterculia* species analyzed, *S. foetida* showed the closest relationship to *S. pexa*, supported by a 100% bootstrap value (Figure 3).

## Discussion and conclusions

The chloroplast genome length among plant species within the same genus is typically conserved (Huang et al. 2024; Park et al. 2024). However, in this study, the complete chloroplast genome of *S. foetida* (162,637 bp) was found to be 1465–2459 bp longer than the genome lengths reported by Liang et al. (2024) for six other *Sterculia* species. This variation may be due to expansions in non-coding regions or the IR regions of the *S. foetida* chloroplast genome; however, further analysis is needed to confirm the underlying cause.

The phylogenetic analysis indicates that the genus *Heritiera* is more closely related to *Sterculia* than to *Firmiana*, consistent with the findings of Wilkie et al. (2006) and Wang et al. (2021). In this study, *S. foetida* and *S. pexa* were shown to share a close evolutionary relationship. Among the eight *Sterculia* species analyzed, *S. brevissima*, *S. nobilis*, *S. euosma*, *S. lanceifolia*, *S. micrantha*, and *S. villosa* have simple leaves, while *S. foetida* and *S. pexa* possess palmately compound leaves. This morphological similarity further supports the formation of a sister clade between *S. foetida* and *S. pexa*.

This study presents the first assembly and annotation of the complete chloroplast genome sequence of *S. foetida*. The resulting genomic data provide a valuable resource for the development of molecular markers, the investigation of evolutionary history, and the reconstruction of phylogenetic relationships within the genus *Sterculia* and the family Malvaceae.

## Supplementary Material

Supplementary Figures.doc

Language editing certificate.pdf

## Data Availability

The genome data supporting the findings of this study are available in the NCBI database (https://www.ncbi.nlm.nih.gov/) under accession number PP417914. The associated BioProject, SRA, and Bio-Sample numbers are PRJNA1095020, SRR28520197, and SAMN40711818, respectively.
