# Supplementary material for: The first complete chloroplast genome sequence of Sterculia foetida Linnaeus (Malvaceae) and a comparative phylogenetic analysis
Source: Mitochondrial DNA B Resour. 2025 Jul 14;10(8):731–5. doi: 10.1080/23802359.2025.2530712 (PMC12261506; doi:10.1080/23802359.2025.2530712)
Supplement: Supplementary Figures.doc [file TMDN_A_2530712_SM2552.doc]

**Figure S1** Overall coverage depth of the chloroplast genome assembly of *Sterculia foetida*
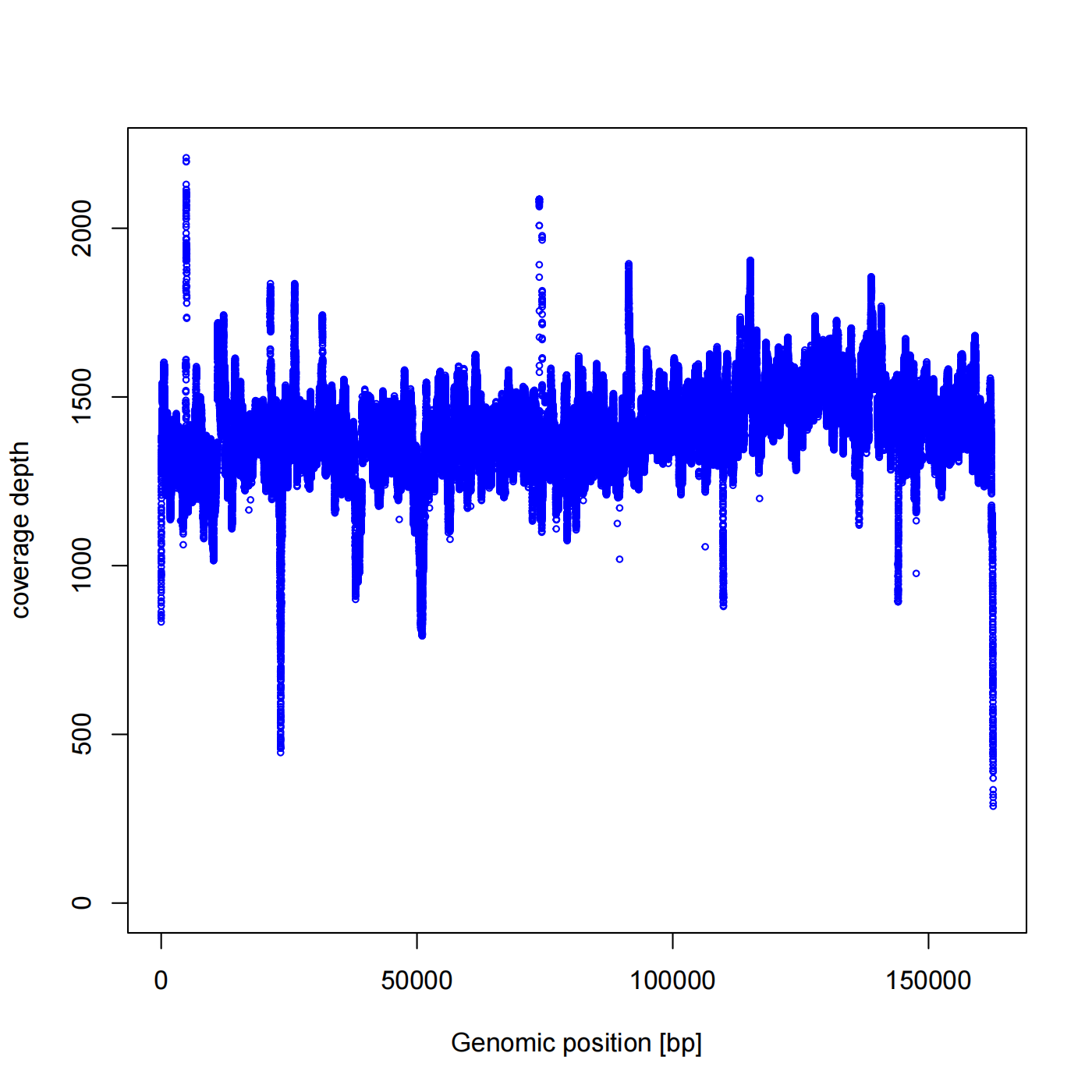
.


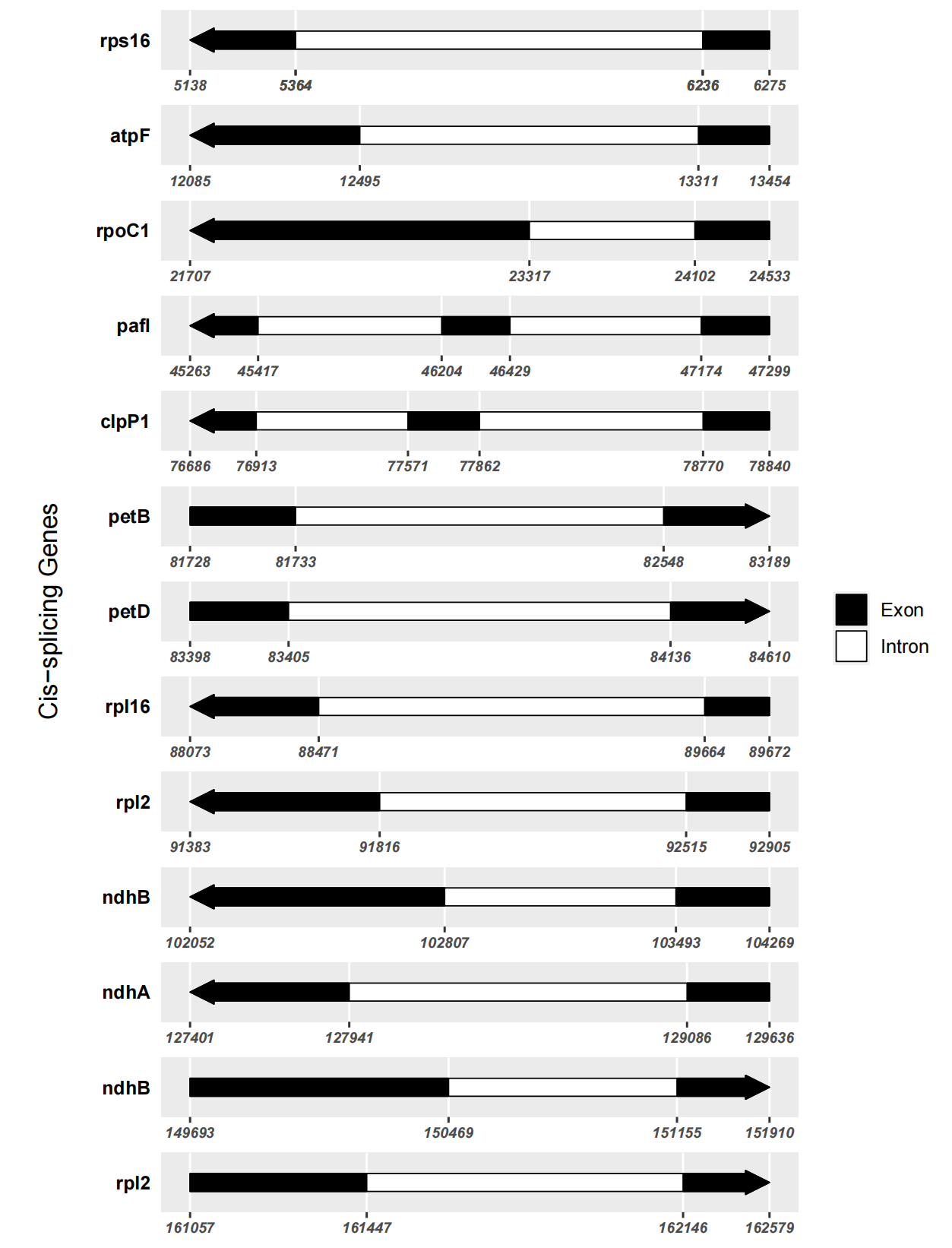


**Figure S2** Schematic map of the cis-splicing genes in the *Sterculia foetida* chloroplast genome.

**Figure S**
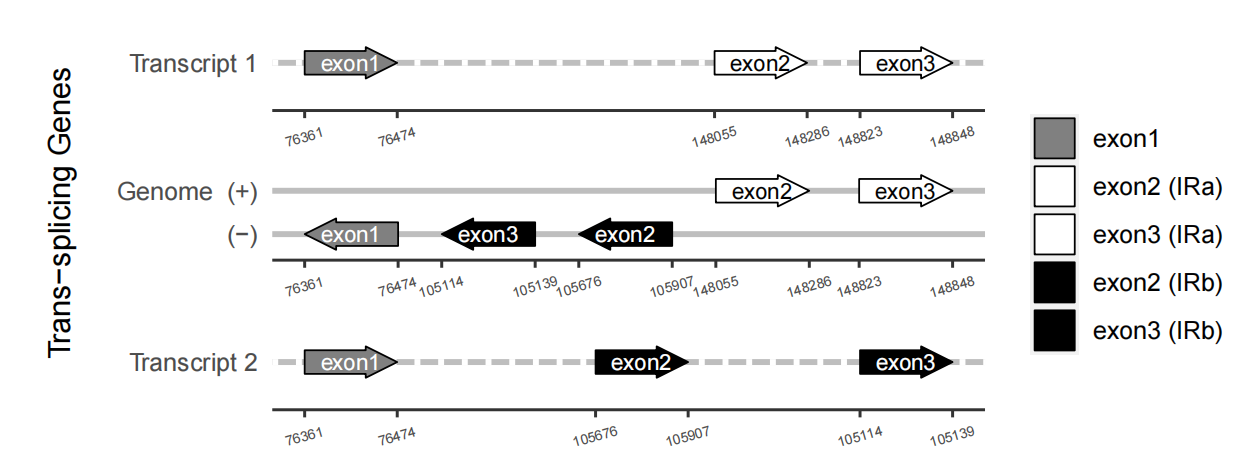
**3** Schematic map of the trans-splicing gene rps12 in the *Sterculia foetida* chloroplast genome.
