# Supplementary material for: The first complete chloroplast genome sequence of Sterculia foetida Linnaeus (Malvaceae) and a comparative phylogenetic analysis
Source: Mitochondrial DNA B Resour. 2025 Jul 14;10(8):731–5. doi: 10.1080/23802359.2025.2530712 (PMC12261506; doi:10.1080/23802359.2025.2530712)
Supplement: Language editing certificate.pdf [file TMDN_A_2530712_SM2551.pdf]

TOPEDIT

TOPEDIT SCIENTIFIC EDITING

CERTIFICATE OF ENGLISH COPYEDITING

This is to certify that the manuscript detailed below has been edited by multiple native English-speaking academic editors in TopEdit. Neither the research content nor the authors' intentions were altered in any way during the editing process. TopEdit guarantees the quality of English grammar, spelling, punctuation, syntax, technical accuracy, and consistency in this manuscript, provided that our editors' corrections and suggestions are accepted and further changes made by the authors are checked by our editors.

Manuscript title

The first complete chloroplast genome sequence of Stercul ia foetida Linnaeus(Malvaceae) and comparative phylogene tic analysis

Date Issued	Certificate Number
06/10/2025	CN 22187-08-0610-01

TopEdit specializes in comprehensive evaluation and academic editing of technical manuscripts, book chapters, grant proposals, and other types of scientific materials. Aiming at breaking language barriers and advancing research publication, we offer four levels of English editing: Proofreading, Copyediting, Line editing, and Developmental editing. Our editorial team comprises professional native English-speaking experts in various academic fields with advanced Ph.D. degrees.

info@topeditsci.com
https://www.topeditsci.com
https://en.topeditsci.com/
